# Supplementary material for: Signal propagation via cortical hierarchies
Source: Netw Neurosci. 2020 Nov 1;4(4):1072–90. doi: 10.1162/netn_a_00153 (PMC7657265; doi:10.1162/netn_a_00153)
Supplement: Supplementary file 1 [file netn-04-1072-s001.pdf]

Vazquez-Rodriguez, B., Liu, Z., Hagmann, P. & Misic, B. (2020). Supporting information for "Signal propagation via cortical hierarchies". *Network Neuroscience*, *4*(4), 1072-1090. https://doi.org/10.1162/netn_a_00153

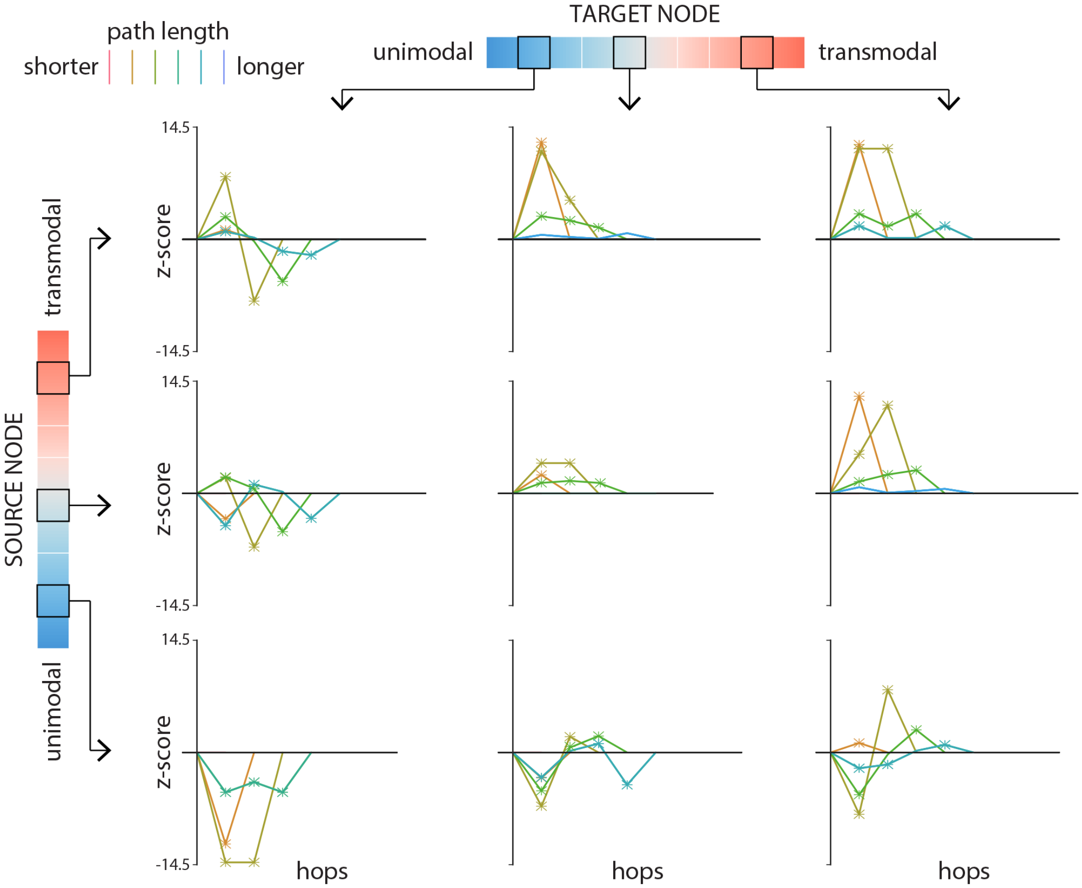

Figure S1. **Path motifs - rewired null model** | For each source-target pair, nodes along the corresponding path are labeled according to their position on the unimodal-transmodal cortical hierarchy. Hierarchy values are binned into 10 equally-sized levels, where level 1 corresponds to unimodal cortex and level 10 corresponds to transmodal cortex. Paths motifs are shown for three levels of source nodes (2, 6 and 9; rows) and three levels of target nodes (2, 6 and 9; columns). Each plot shows the z-score of the mean path motif relative to a population of rewired networks with preserved degree sequence and cost [11]. Path position (hop) is shown on the x-axis. Paths are stratified according to their length, such that warmer colours indicate shorter paths and colder colours indicate longer paths. Points denoted by asterisks indicate $p < 0.05$ (FDR-corrected).

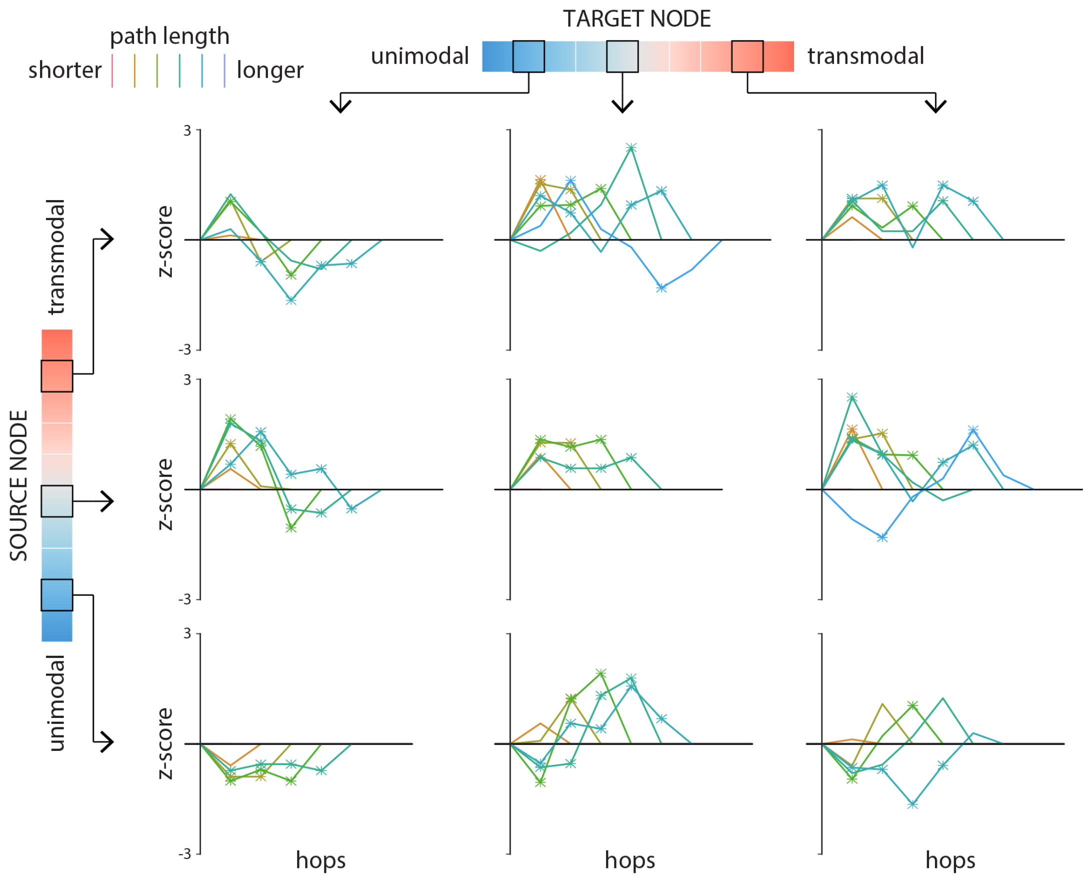

Figure S2. **Path motifs - spin null model** | For each source-target pair, nodes along the corresponding path are labeled according to their position on the unimodal-transmodal cortical hierarchy. Hierarchy values are binned into 10 equally-sized levels, where level 1 corresponds to unimodal cortex and level 10 corresponds to transmodal cortex. Paths motifs are shown for three levels of source nodes (2, 6 and 9; rows) and three levels of target nodes (2, 6 and 9; columns). Each plot shows the z-score of the mean path motif relative to a label-permuting null model that preserves the spatial autocorrelation of hierarchical labels [2, 76]. Path position (hop) is shown on the x-axis. Paths are stratified according to their length, such that warmer colours indicate shorter paths and colder colours indicate longer paths. Points denoted by asterisks indicate $p < 0.05$ (FDR-corrected).

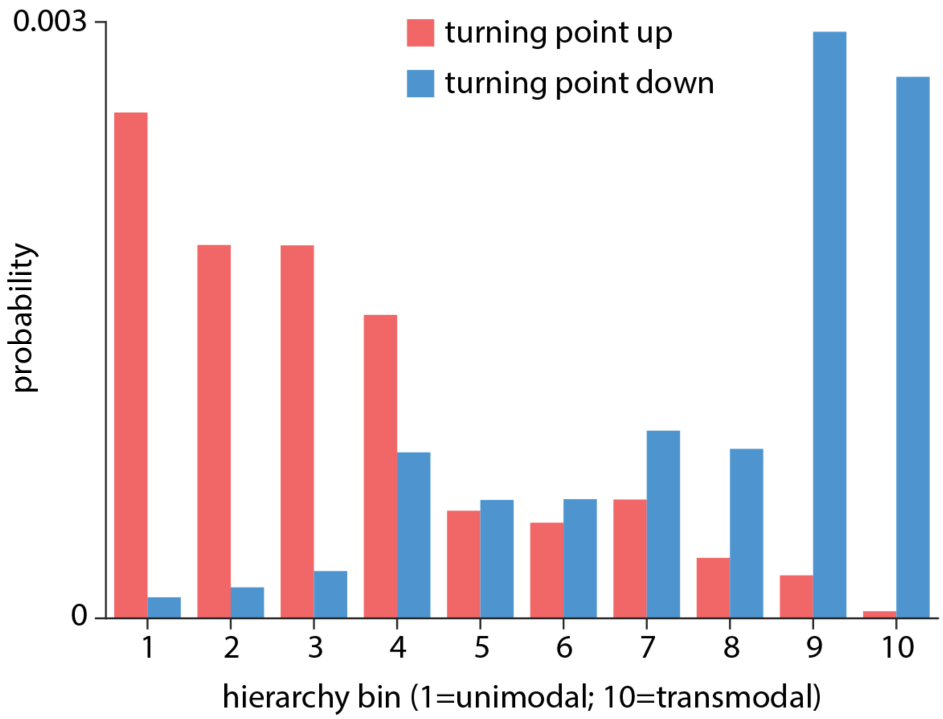

Figure S3. **Turning point probabilities across the unimodal-transmodal hierarchy** | The distribution of turning points, stratified by hierarchical label. The probability of being a turning point is related to a node's position in the hierarchy, but importantly, the histogram shows that there exist turning points of both kinds at all levels of the hierarchy, with an approximately equal mix in the middle (bins 5-7).

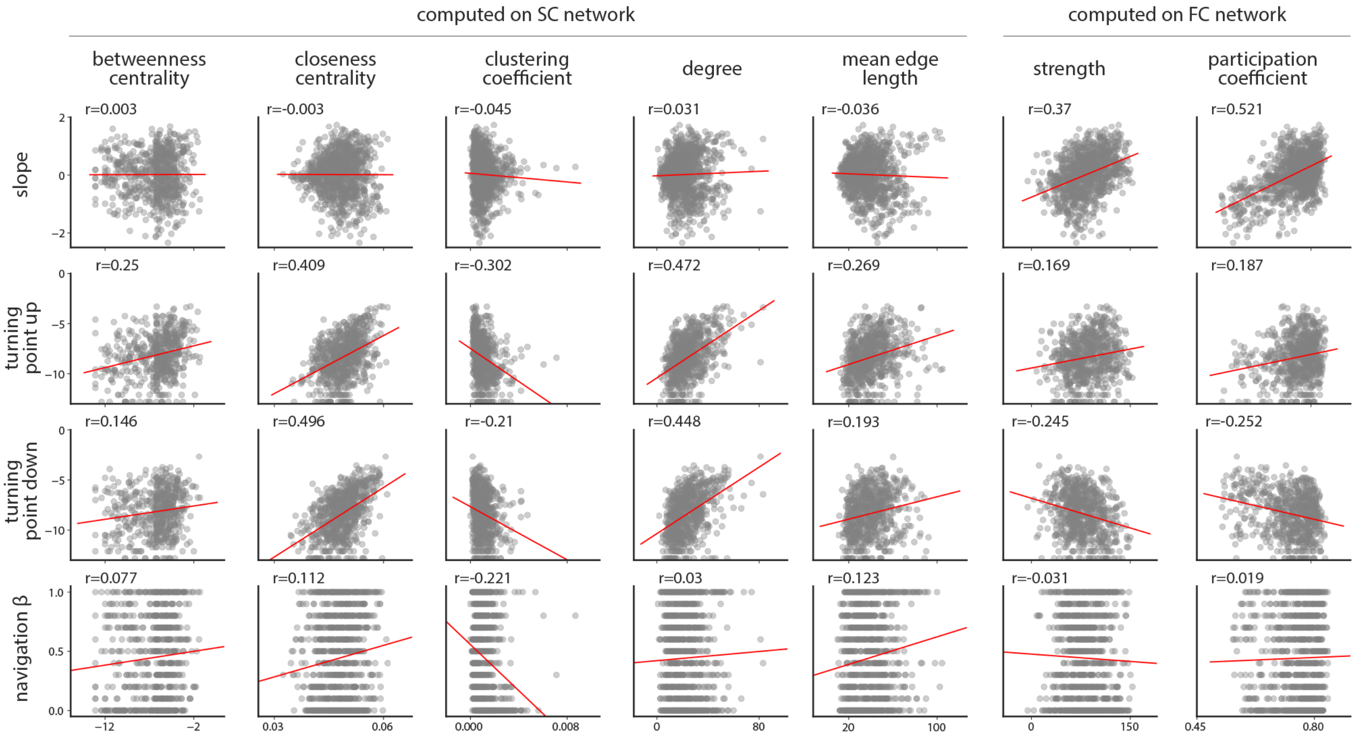

Figure S4. **Relating path motif measures with graph properties** | The present report derives four node-level dependent variables (mean slope, turning up/down point probability and navigation preference). To assess the extent to which these measures are related to simpler graph properties, we correlate them with node-level features from same structural and functional connectivity matrices. Path motif measures are shown in the rows and graph measures are shown in the columns. The first five graph measures (betweenness, closeness, clustering, degree and mean edge length) are computed on the structural network; the last two graph measures (strength and participation) are computed on the functional network. Edge length refers to physical length and is measured in mm. Participation coefficient is computed with respect to the intrinsic network partition provided by Yeo and colleagues [81]. The two turning point measures and betweenness centrality are log transformed. Relationships are reported in terms of Pearson correlation coefficients.

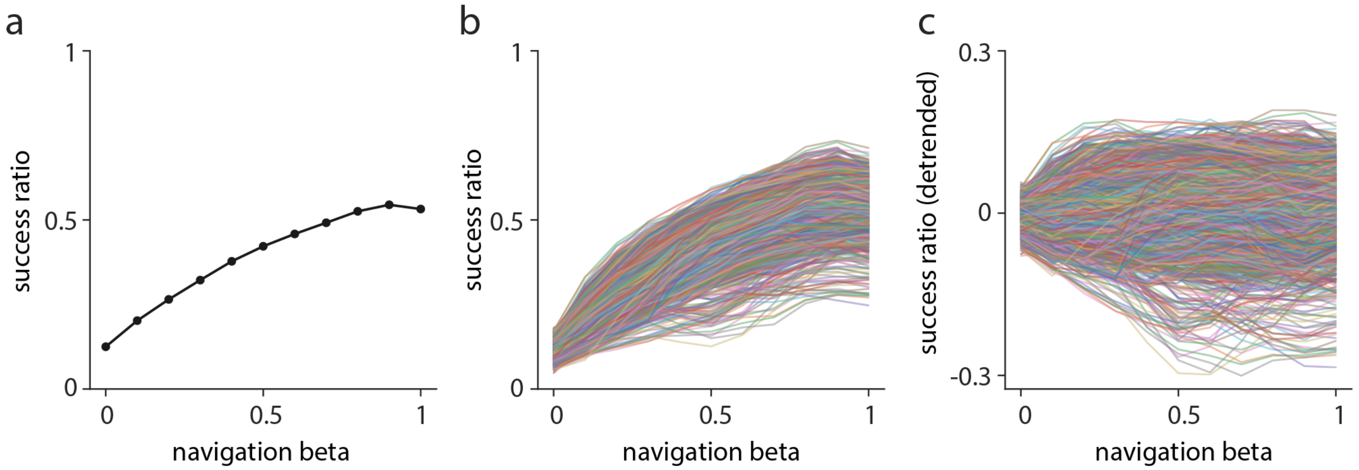

Figure S5. **Navigation via space and hierarchy** | To assess the use of spatial and hierarchical information to navigate, we derive the proportion of shortest paths successfully recovered (success ratio) as a function β, which tunes preference for hierarchy ($\beta = 0$) vs spatial ($\beta = 1.0$) information. (a) When the same β is imposed on all nodes, there is a stronger preference for spatial navigation, with the optimal $\beta = 0.9$. (b) Fitting β separately for each source node, we observe the same preference for spatial navigation, but also substantial variability across nodes. (c) To ask whether some nodes additionally benefit from hierarchical information, above and beyond spatial information, we detrend the curves and focus on the residual success ratios.
